# A New Era of Minimally Invasive Surgery: Progress and Development of Major Technical Innovations in General Surgery Over the Last Decade

**DOI:** 10.1055/s-0037-1608651

**Published:** 2017-11-09

**Authors:** Manjunath Siddaiah-Subramanya, Kor Woi Tiang, Masimba Nyandowe

**Affiliations:** 1 Department of Surgery, Logan Hospital, Brisbane, Queensland, Australia; 2 Department of Medicine, Griffith University, Queensland, Australia; 3 Department of Medicine, University of Queensland, Queensland, Australia; 4 Department of Surgery, Townsville Hospital, Townsville, Queensland, Australia

**Keywords:** laparoscopy, innovation, minimally invasive surgery, keyhole surgery, natural orifice transluminal endoscopic surgery, single-incision laparoscopic surgery

## Abstract

Minimally invasive surgery (MIS) continues to play an important role in general surgery as an alternative to traditional open surgery as well as traditional laparoscopic techniques. Since the 1980s, technological advancement and innovation have seen surgical techniques in MIS rapidly grow as it is viewed as more desirable. MIS, which includes natural orifice transluminal endoscopic surgery (NOTES) and single-incision laparoscopic surgery (SILS), is less invasive and has better cosmetic results. The technological growth and adoption of NOTES and SILS by clinicians in the last decade has however not been uniform. We look at the differences in new developments and advancement in the different techniques in the last 10 years. We also aim to explain these differences as well as the implications in general surgery for the future.

Health care innovation, including ones in the field of minimally invasive surgery (MIS), can be defined as a dynamic and continuous process involving the introduction of a new technology or technique that initiates a change in practice. 1 There have been constant innovations to improve MIS since its emergence in the early 1980s, although the basic concepts have changed little. They include technological innovations in instruments used, such as laparoscopic instruments and sutures or MIS-associated technology, such as surgical robotics, image guidance systems, natural orifice transluminal endoscopic surgery (NOTES) and single-incision laparoscopic surgery (SILS).

There are distinct patterns of growth, development, and innovations in MIS since the early 1980s represented by the number of patent applications and literature publications, with each of these patterns containing technologies with unique characteristics. 2 The first growth pattern was in relation to novel surgical instruments and sutures to complement MIS. This growth shows a peak in the mid-1990s and then again in the mid-2000s. The peak in the 1990s was mainly in relation to basic laparoscopic instruments when the concept was introduced, and steps are taken to popularize. 1 A new growth spurt with instruments was seen again in the mid-2000s as the laparoscopic procedures became well established and widely used necessitating more ergonomics instruments.

The second growth phase noted is with regards to surgical robotics and image guidance. Their growth shows gradual and exponential patterns starting in the mid-1990s throughout 2000, and beyond. 2 The reason for this growth pattern is probably multifactorial. These technologies, in spite of numerous complex engineering challenges, have demonstrated continued development to keep up with the clinical demand. Continued development of robotic technology resulted in third generation surgical robots. These technologies also serve to expand the practice of MIS rather than just providing necessary tools for the MIS. This is evident in increased usage of robotics in various operations, sometimes even acting as a complementary technology for an existing method, such as SILS.

The latest and the third growth phase was noted in the late 2000s in relation to NOTES and SILS with its inception in the mid-2000s, peaking soon after that. Although the popularity of NOTES plateaued in late 2000, SILS has continued to receive interest. The reason for this plateau with NOTES is partly due to dwindling of innovation and interest in the technique, and partly due to the profound difference between innovators and adopters. Conversely, SILS may likely have a brighter future owing to easier access to technology and instrumentation, specialist to mainstream practice, and possibly with increasing popularity of robotics, which may complement SILS. 2

One of the biggest advances in MIS in the last decade is in the field of robotic surgery. Robotics was introduced for surgery in civilian hospitals in the early 1990s, although it was initially used in the military environment performing surgeries in the 1970s. 3 Robotics combined with computer science has been able to augment surgeon's skills to achieve greatly improved accuracy and precision in complex surgery. Ever improving technology in optics and computer science has introduced virtual reality (VR) and three dimensional (3D) to operating rooms. 4 5 This allows for the development of patient-specific models enabling planning and practice of complex surgery on VR platform before performing the actual surgery. The 3D virtual model improves the mental representation of anatomical details, which could be underestimated with two-dimensional visualization platform that is more commonly used currently in operating suites.

Robotic surgery has evolved immensely since the initial operating room version Zeus (Computer Motion). Newer models of surgical robots, Da Vinci (Intuitive Surgical), feature compact mobile platforms, multiple operating arms, and superior surgeon's console equipped with surgeon- piloted stereotactic 3D immersive and ergonomic handles intuitive to human hand movements providing improved dexterity. Other robotic platforms have been approved and are in various stages of development and introduction to the surgical market. They claimed to produce small robotic platforms with better maneuverability, more user-friendly in constricted spaces, provide force feedback and eye tracking capabilities. The eye tracking technology works with the aid of a camera mounted on an eyewear, which could track the surgeons' eye movements and move the scope inside the patient accordingly. Some of the examples are Amadeus Composer (Titan Medical Inc.) from Canada and TELELAP Alf-X (TransEnterix) from Italy. 3

The application of robotic surgery potentially is much wider than just restricted to operating theater where the robot is physically located. The current platform enables remote access enabling telesurgery, without the need for the surgeon to be present physically in the operating theater. One such event was a surgical operation performed in Strasbourg (France) by surgeons in New York (United States), which became a milestone in global telesurgery. 6 7 Furthermore robotic surgery experiments have been performed in a weightless environment. 8 9 10 Considering the current quality and speed of web-based transmission of signals, it would make remote surgery on any facility orbiting the earth, such as international space station, possible. Currently, it would require more advanced telecommunication for surgeries at a distance further from the moon. 11

The role of robotic surgery compared with laparoscopic surgery is debatable, mainly due to high cost and equivocal surgical outcome. In spite of that robotic surgery remained appealing to health care organizations and surgeons with a passion for cutting-edge technology. Astronomical cost while a disadvantage may change with improved platforms that are easier and quicker to set up, which improves further with experience, and lower cost with vanishing monopoly in the production of surgical robots.

Robotic surgery in the peritoneal cavity has been investigated fairly extensively, and the technology has proven to be of benefit in selected operations. Robots have been used in colorectal surgery for over 10 years. 12 A systematic review concluded that robotic surgery had a reduced rate of conversion rate to open in rectal surgery. However, no difference was found in duration of surgery, morbidity, and oncological outcomes in either rectal or colonic surgery. 13 When it comes to upper gastrointestinal surgery, especially oncological surgeries, such as gastrectomy and esophagectomy, there is very little benefit in the usage of robots over laparoscopic surgery. 14 15 16 On the other hand, some definite benefit has been shown in benign upper gastrointestinal surgeries where precision is of utmost importance, such as Heller myotomy where it reduces perforation rates. 17 In the field of bariatric surgery, robots aid in reducing the steep learning curve in Roux-en-Y gastric bypass (RYGBP) by making intracorporeal suturing easier and eliminates the use of staplers, potentially proving to be cost-effective compared with laparoscopic RYGBP. 18 19

In hepatobiliary surgery, robotic surgeries have not demonstrated a clear superiority compared with laparoscopic surgery. 20 However, there is some evidence that it may be useful in achieving higher rates of radical R0 resection in pancreatic cancers. 21 Currently, there is a paucity of experience regarding liver resection to draw any major conclusions. 22

Another significant innovation in the last decade is NOTES, described by some as perhaps the most significant innovation in surgery since Phillipe Mouret of France performed the first laparoscopic cholecystectomy in 1987. 23 Although, it was Kalloo et al in 2004 that brought the technique into the spotlight. 24 It appears to be a stepwise progression from endoscopic mucosal resection before anyone dared to breach the muscular layer intentionally. This novel technique was a result of a harmonious union between gastroenterologists and surgeons in America in early 2000. Since then several NOTES procedures have been performed using mainly stomach, rectum, and vagina as the portal of entry into the peritoneal cavity. NOTES was also the first “scar less,” surgical technique introduced to the public and their perception, initially at least, was in favor of this technique. 25

There are several barriers to NOTES. Some of them include difficulty in the closure of enterotomy, anastomotic techniques, spatial orientation, long learning curve, lack of triangulation of instruments, control of hemorrhage, and prevention of the transluminal spread of infection. At the same time, there are advantages associated with NOTES. They include no scars, less external pain, lower cost, an alternative to the laparoscopic procedure in a patient not suitable for laparoscopy and it even could act as a complementary technology to laparoscopic surgery and avoid major resections.

Unfortunately, over the last decade NOTES encountered more problems than solutions that the industries are still trying to correct. Therefore it has hit a plateau in its popularity, and usage. 2 Comparable results were noticed in the first nonrandomized trial to be published comparing diagnostic laparoscopy and transgastric peritoneoscopy after careful selection of patients. 26 This study demonstrated the usefulness of NOTES while testing its specific aspects but does not improve the safety of NOTES in general.

While the closure of an enterotomy remains a huge issue, access and triangulation are fundamental to the success of MIS. Some surgeons have endeavored to address these issues. Combining laparoscopy with NOTES has been suggested and trialed to improve insufflation, orientation, retraction, instrument navigation, and solid organ manipulation. 27 Another novel technique: dual access NOTES has been proposed and tested to improved handling, orientation, and maneuverability (e.g., rectal and gastric). 28 29 However, dual access doubles the risk of contamination, infection, and luminal closure difficulties. Various companies engineered different devices address problems associated with the closure of enterotomy. They range from simple endoscopic dexeclips used to close enterotomies as large as 4 cm to purse string applicators used to close gastric incisions and g-prox (USGI Medical) tissue grasper. 30 31 32 Some have only been used in animal models.

Developments in VR, stereoscopic 3D cameras, and augmented reality (AR) camera are some of the areas worth following in the years to come. Conventional cameras are two-dimensional and lack depth perception. At present da Vinci robotic camera has 3D visualization capability but extending that technology to the laparoscopic camera has had its challenges. Although Olympus has introduced a video-assisted camera capable of 3D visualization, it lacks the resolution that robotic 3D camera provides. Some research groups have reported developing AR visualization for laparoscopic cameras. 33 34 Here, the preoperative images are registered in a stationary format, which is then superimposed on the available intraoperative images from the laparoscopic camera. However, the surgeon normally constantly manipulates the tissues and organs, in reality, making the abovementioned model less useful. Upcoming technologies claim that they could reconstruct preoperative images in real-time according to patient's body shape. 35

Another technology worth mentioning is a laparoscopic ultrasound (LUS), which is two-dimensional with the images displayed on a separate monitor that unfortunately forces the surgeons to take their eyes off the organ or laparoscopic screen. With the combination of LUS and AR technology in a stereoscopic 3D camera one can now view the organ that is subjected to ultrasound and its abnormalities in real-time directly on the organ itself and make surgical decisions for accurate dissection with precise movements, so that resection margins are kept to minimum but sufficient and safeguarding the surrounding structures that may not be visible in 2D view. 36

## Conclusion

Our future consists of exciting, new emerging technologies, which may make MIS even more efficient, exciting, and safe. The possibilities are limitless, and we await more innovations to enable more sensible applications of different surgical techniques and instruments.
